# Quantitative analysis of histone exchange for transcriptionally active chromatin

**DOI:** 10.1186/2043-9113-1-17

**Published:** 2011-07-07

**Authors:** Stephanie D Byrum, Sean D Taverna, Alan J Tackett

**Affiliations:** 1 University of Arkansas for Medical Sciences, 4301 West Markham Street, Little Rock, Arkansas 72205, USA; 2 Johns Hopkins School of Medicine, 855 North Wolfe Street, Baltimore, Maryland 21205, USA

**Keywords:** cross-linking, histone, post-translational modification, chromatin, affinity purification

## Abstract

**Background:**

Genome-wide studies use techniques, like chromatin immunoprecipitation, to purify small chromatin sections so that protein-protein and protein-DNA interactions can be analyzed for their roles in modulating gene transcription. Histone post-translational modifications (PTMs) are key regulators of gene transcription and are therefore prime targets for these types of studies. Chromatin purification protocols vary in the amount of chemical cross-linking used to preserve *in vivo *interactions. A balanced level of chemical cross-linking is required to preserve the native chromatin state during purification, while still allowing for solubility and interaction with affinity reagents.

**Findings:**

We previously used an isotopic labeling technique combining affinity purification and mass spectrometry called transient isotopic differentiation of interactions as random or targeted (transient I-DIRT) to identify the amounts of chemical cross-linking required to prevent histone exchange during chromatin purification. New bioinformatic analyses reported here reveal that histones containing transcription activating PTMs exchange more rapidly relative to bulk histones and therefore require a higher level of cross-linking to preserve the *in vivo *chromatin structure.

**Conclusions:**

The bioinformatic approach described here is widely applicable to other studies requiring the analysis and purification of cognate histones and their modifications. Histones containing PTMs correlated to active gene transcription exchange more readily than bulk histones; therefore, it is necessary to use more rigorous *in vivo *chemical cross-linking to stabilize these marks during chromatin purification.

## Introduction

Eukaryotic genomes are highly organized into transcriptionally active (euchromatic) and silent (heterochromatic) chromatin regions. Conversion of chromatin between the two major forms is regulated in part through interactions between chromatin-modifying enzymes and nucleosomes. Nucleosomes are the fundamental unit of chromatin and consist of approximately 147 base pairs of DNA wrapped around an octameric core of the histones H2A, H2B, H3, and H4 [1]. Chromatin structure plays a key role in the regulation of gene activity and its mis-regulation is a theme characteristic of many types of disease and cancer [1]. The N-terminal tails of histones, which protrude outside of the nucleosome core [2], are subject to many sites and types of post-translational modifications (PTMs), which, in turn, help regulate biological processes through altering nucleosome stability or the function of chromatin-associated complexes [3,4]. For example, acetylation of histone lysine residues on the N-terminal tail has been correlated to active gene transcription either by countering the negative charge of the DNA backbone, or through the recruitment or stabilization of bromodomain-containing proteins [3,5,6].

A major emphasis in the field of chromatin biology is the understanding of how histone PTMs and protein-protein interactions are associated with specific gene loci to regulate gene transcription. Current technologies like ChIP (chromatin immunoprecipitation), affinity purification of protein-histone complexes for proteomic analysis, and more recent technology that allows for the purification of chromosome sections for proteomic analysis are used to study protein interactions on chromosomes [7-10]. One pitfall of these technologies is the challenge of purifying cognate histones (i.e., preserving the *in vivo *associated histones during isolation of chromatin). To overcome this pitfall, we have previously reported how to monitor and prevent dynamic exchange of histones during chromatin purification [11]. *In vivo *chemical cross-linking reagents, such as formaldehyde, can be used to prevent histone exchange during the purification of chromatin sections [12]. However, there is a balanced level of chemical cross-linking needed to trap protein-protein and protein-DNA interactions, while still allowing for the solubility of chromatin for purification and access of affinity reagents [12].

We have recently published a quantitative approach using I-DIRT, an isotopic labeling technique utilizing affinity purification and mass spectrometry, to measure levels of histone exchange in purified chromatin sections [11]. Here we describe a bioinformatic analysis, which expands on this published work, reporting the significance of proper cross-linking to capture histones with transcription activating PTMs during chromatin purification. In this work, we are able to gain new insights into the dynamic exchange of histones and post-translationally modified histones.

## Experimental Methods

Detailed methods are described in Byrum et al. 2011. Briefly, *Saccharomyces cerevisiae HTB1::TAP-HIS3 BY4741 *(Open Biosystems) cells grown in isotopically light media and cells from an arginine auxotrophic strain (*arg4::KAN BY4741*, Open Biosystems) cultured in isotopically heavy media (^13^C_6 _arginine) were grown to midlog phase (3.0 × 10^7 ^cells/mL) and cross-linked using either 0%, 0.05%, 0.25%, or 1.25% formaldehyde (FA). The cells were harvested, mixed 1:1 by cell weight (isotopically light cells: heavy cells), and lysed under cryogenic conditions. The cell powder was resuspended in affinity purification buffer (20 mM HEPES pH 7.4, 300 mM NaCl, 0.1% tween-20, 2 mM MgCl_2_, and 1% Sigma fungal protease inhibitors) and the DNA sheared to ~1 kb sections. Small chromatin sections containing TAP tagged H2B histones were affinity purified on IgG-coated Dynabeads and the eluted proteins were resolved with a 4-20% Tris-Glycine gel. Following colloidal Coomassie-staining, histone gel bands were excised, trypsin digested, and tryptic peptides were subjected to tandem mass spectrometric analysis with a coupled Eksigent NanoLC-2D and Thermo LTQ-Orbitrap mass spectrometer [12]. The histone purification experiments were performed in triplicate.

The isotopically light and heavy arginine containing histone peptides were identified using a Mascot (version 2.2.03) database search. Peptide identification can be made with mass spectrometric database searching software other than Mascot with equivalent results. The search parameters included: precursor ion tolerance 10 ppm, fragment ion tolerance 0.6 Da, fixed modification of carbamidomethyl on cysteine, variable modification of oxidation on methionine and acetyl on lysine, and 2 missed cleavages possible with trypsin. The Mascot results were uploaded into Scaffold 3 (version 3.00.01) for viewing the proteins and peptide information. A false discovery rate of 1% was used as the cut off value for arginine containing histone peptides. The monoisotopic peak intensity (I) values for each arginine containing peptide were extracted using Qual Browser (version 2.0, Thermo). The percent light for each peptide was calculated as I_L_/(I_L _+ I_H_). The average of all peptides identified for each percentage of cross-linking was calculated along with the standard error. The number of unique identified peptides was: bulk H3 (26, 14, 9, and 8), H3K9acK14ac (7, 4, 8, and 8), bulk H4 (25, 8, 8, and 13), and H4K12acK16ac (7, 4, 5, and 3) for 0%, 0.05%, 0.25% and 1.25% FA, respectively. Percent light peptide reported here differs from the Byrum et al report [11] as we have separated PTM containing and unmodified peptides in the current report.

## Results and Discussion

**Figure 1:**
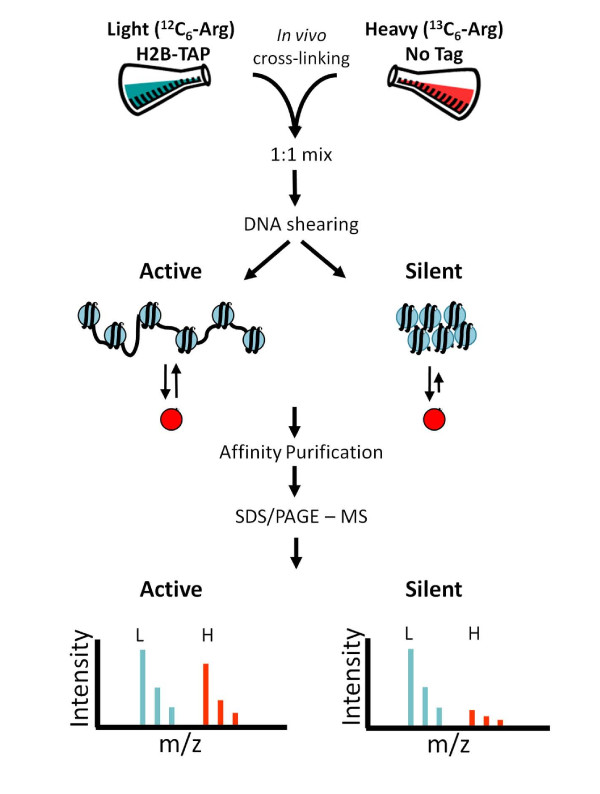
**Quantitative Analysis of histone exchange**. *S. cerevisiae *H2B-TAP cells were grown in isotopically light media (^12^C_6_-Arg) while an arginine auxotrophic strain was grown in isotopically heavy (^13^C_6_-Arg) media. Cultures were chemically cross-linked with formaldehyde, harvested independently, mixed 1:1 by cell weight, and cryogenically co-lysed. Chromatin was sheared to ~1 kb and affinity purified on IgG coated Dynabeads. Histones were resolved by SDS-PAGE and the percent light peptides were measured by mass spectrometry. Depending on the level of *in vivo *cross-linking, histones will dissociate and re-associate with the purified chromatin. This exchange can be monitored by measuring the incorporation of isotopically heavy histones (red circles). Actively transcribing chromatin is more loosely packaged and will undergo histone exchange more readily. Silent chromatin is more densely packaged and is less likely to undergo histone exchange.

The potential roles histone modifications play in regulating gene transcription and the recruitment of protein complexes to specific gene loci have made them attractive therapeutic targets for a variety of diseases including cancer. In order to preserve and study histone PTMs that occur on specific sites of chromatin, histone exchange must be prevented during the chromatin purification process. We previously utilized transient I-DIRT technology to investigate the level of chemical cross-linking with formaldehyde necessary to prevent histone exchange during chromatin purification [11]. Here, we have performed new bioinformatic analyses that reveal differential exchange rates for histones containing PTMs correlated to active gene transcription. As shown in Figure 1 and detailed in the Experimental Methods section, isotopically light histones were isolated via a TAP tag on H2B in the presence of an equivalent amount of isotopically heavy histones. The exchange of histones (i.e., the incorporation of isotopically heavy histones during the isolation of isotopically light histones) was followed with mass spectrometry.

**Figure 2:**
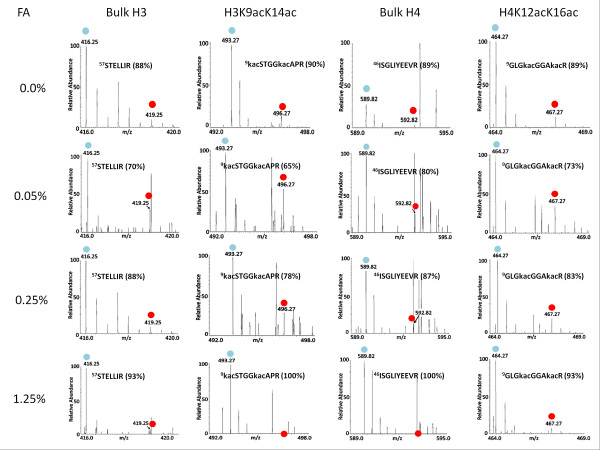
**Mass spectra of PTM-containing histone peptides**. Mass spectra were collected with an Orbitrap mass analyzer for doubly charged peptides from bulk histone H3, H3K9acK14ac, bulk histone H4, and H4K12acK16ac. Blue circles indicate the isotopically light peak while red circles indicate the isotopically heavy peak. The percent isotopically light is shown in parentheses and *in vivo *formaldehyde (FA) cross-linking percentages are listed.

**Figure 3:**
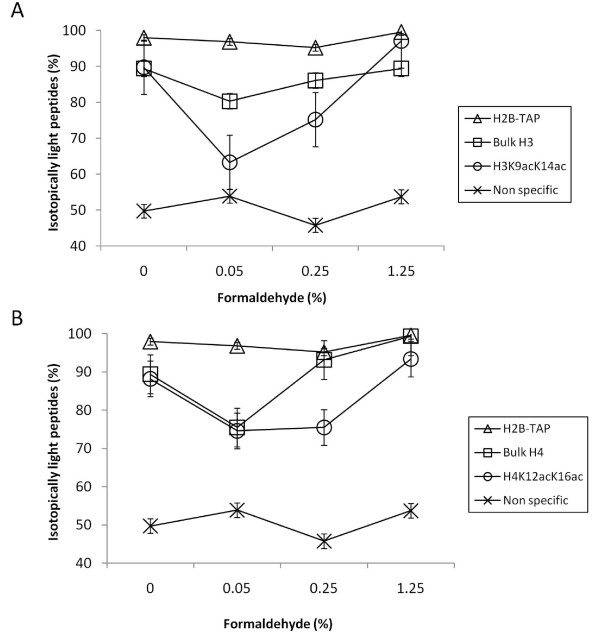
**Histone exchange occurs more readily in chromatin containing transcription activating PTMs**. (A) The average and standard error of isotopically light arginine containing peptides for bulk H3, H3K9acK14ac, H2B-TAP, and 15 non-specifically associating proteins are plotted as a function of formaldehyde cross-linking. (B) Plot of bulk H4, H4K12acK16ac, H2B-TAP, and 15 non-specific proteins as a function of formaldehyde cross-linking. Levels approaching 100% light peptides indicate minimal histone exchange while levels at ~50% light peptides reflect rapid exchange.

Mascot analysis of the mass spectrometric data obtained from H2B-TAP cells treated with increasing amounts of formaldehyde identified lysine acetylation marks on histone H3 lysine 9 and lysine 14 (H3K9acK14ac) and histone H4 lysine 12 and lysine 16 (H4K12acK16ac). H3K9acK14ac and H4K12acK16ac are reported marks of active gene transcription, as is acetylation at many other histone lysines [5,6,13,14]. Representative mass spectra of bulk H3, H3K9acK14ac, bulk H4, and H4K12acK16ac peptides for each percentage of cross-linking are shown in Figure 2. The average percent light of all peptides identified for each histone is plotted in Figure 3. Percent light values approaching 100% light peptides indicate minimal histone exchange during purification while those near 50% light peptides reflect rapid exchange. Peptides from the H2B-TAP control were ~100% light at all formaldehyde concentrations tested. The reason that the H2B-TAP peptides are ~100% light is that the TAP tagged version of H2B is only expressed in the strain grown in isotopically light media. This isotopically light TAP tagged version of H2B migrates slower in SDS-PAGE due to the ~20 kDa molecular mass addition of the TAP tag; thus, excision of this band on the gel is exclusively for isotopically light H2B-TAP as all other histones migrate further in the gel. Non-specific proteins co-enriching with H2B-TAP have ~50% light peptides, reflecting the mixing of isotopically light and heavy cultures prior to purification. Without cross-linking, ~10% histone exchange during purification was observed (Figure 3). As reported previously in Byrum et al 2011, mild cross-linking at 0.05% actually increased the observed level of histone exchange during purification, which was not observed at elevated levels of cross-linking. We predict that cross-linking more readily stabilizes densely packaged areas of chromatin like heterochromatin, while leaving less densely packaged regions less stable. In accordance, as densely packaged chromatin becomes more heavily cross-linked, it becomes less represented in the analysis due to less efficient DNA shearing and solubility for purification. At a low level of formaldehyde (0.05%), histone H3K9acK14ac peptides are closer to non-specific percent light indicating rapid histone exchange; however, bulk histone H3 is ~80% light. This reveals that histones modified with activating transcription marks exchange more readily than histones without the transcription activating marks. This likely reflects the less densely packaged euchromatin that is more transcriptionally active. At 0.25% formaldehyde, acetylated histone H3K9acK14ac showed greater exchange compared with bulk H3; however, they both have increased percent light peptides indicating the minimization of exchange with increasing formaldehyde cross-linking. Bulk histone H4 and H4K12acK16ac had similar percentages of light peptides at 0.05% formaldehyde; however, acetylated H4 showed more exchange than bulk H4 at 0.25% formaldehyde. All bulk and acetylated peptides had ~100% light peptides at 1.25% formaldehyde, which indicated that the histones are minimally exchanged. Therefore, 1.25% formaldehyde is sufficient to prevent exchange of histones containing PTMs correlated to gene transcription during our purification of chromatin sections. The percent of formaldehyde cross-linking is specific for yeast synthetic media as other medias require different levels depending on their amine or cross-linking moiety content.

## Conclusions

We have previously published the application of I-DIRT technology to determine the level of histone dissociation/re-association during chromatin purification [11]. In this report, we have applied additional bioinformatic analyses to study the dynamics of exchange for histones containing transcription activating PTMs. As demonstrated in the histone exchange analysis shown in Figure 3, we show that chromatin marked for gene transcription is susceptible to the loss of histones during purification and therefore requires sufficient levels of *in vivo *chemical cross-linking to preserve the native chromatin composition. The technique reported in Byrum et al. 2011 and further analyzed here is relevant for a variety of genome-wide studies, and should be considered when preservation of *in vivo *chromatin content is essential for functional analyses, especially when examining transcriptional processes.

## Abbreviations

I-DIRT: (isotopic differentiation of interactions as random or targeted); FA: (formaldehyde); ChIP: (chromatin immunoprecipitation); PTMs: (post-translational modifications)

## Competing interests

The authors declare that they have no competing interests.

## Authors' contributions

SDB carried out the experiments, data analysis, and drafted the manuscript. SDT and AJT conceived of the study and participated in its design and coordination. AJT helped to draft the manuscript. All authors read and approved the final manuscript.
